# Transcriptomics and Physiological Analyses Reveal Changes in Paclitaxel Production and Physiological Properties in *Taxus cuspidata* Suspension Cells in Response to Elicitors

**DOI:** 10.3390/plants12223817

**Published:** 2023-11-10

**Authors:** Zirui Zhao, Yajing Zhang, Wenlong Li, Yuanhu Tang, Shujie Wang

**Affiliations:** College of Biology and Agricultural Engineering, Jilin University, Changchun 130022, China

**Keywords:** *Taxus cuspidata* suspension cells, elicitors, paclitaxel, physiological responses, transcriptomics

## Abstract

In this research, the cell growth, physiological, and biochemical reactions, as well as the paclitaxel production, of *Taxus cuspidata* suspension cells after treatment with polyethylene glycol (PEG), cyclodextrin (CD), or salicylic acid (SA) (alone or in combination) were investigated. To reveal the paclitaxel synthesis mechanism of *T. cuspidata* suspension cells under elicitor treatment, the transcriptomics of the Control group and P + C + S group (PEG + CD + SA) were compared. The results show that there were no significant differences in cell biomass after 5 days of elicitor treatments. However, the content of hydrogen peroxide (H_2_O_2_) and malondialdehyde (MDA), and the activities of phenylalanine ammonia-lyase (PAL) and polyphenol oxidase (PPO) after elicitor combination treatments were decreased compared with the single-elicitor treatment. Meanwhile, the antioxidant enzyme activity (superoxide dismutase (SOD), catalase (CAT), and peroxidase (PO)) and the contents of soluble sugar and soluble protein were increased after combination elicitor treatments. Additionally, the paclitaxel yield after treatment with the combination of all three elicitors (P + C + S) was 6.02 times higher than that of the Control group, thus indicating that the combination elicitor treatments had a significant effect on paclitaxel production in *T. cuspidata* cell suspension culture. Transcriptomics analysis revealed 13,623 differentially expressed genes (DEGs) between the Control and P + C + S treatment groups. Both GO and KEGG analyses showed that the DEGs mainly affected metabolic processes. DEGs associated with antioxidant enzymes, paclitaxel biosynthesis enzymes, and transcription factors were identified. It can be hypothesized that the oxidative stress of suspension cells occurred with elicitor stimulation, thereby leading to a defense response and an up-regulation of the gene expression associated with antioxidant enzymes, paclitaxel synthesis enzymes, and paclitaxel synthesis transcription factors; this ultimately increased the production of paclitaxel.

## 1. Introduction

*Taxus cuspidata* is a valuable, slow-growing evergreen conifer belonging to the yew family that originated during the Tertiary glacial period and mainly grows in the northeast of China, the Korean Peninsula, Japan, and the Russian Far East [1,2]. The needles and branches of *T. cuspidata* have diuretic and purgative properties, and were employed to treat cholera and typhoid in ancient China. Today, antitumor, anti-inflammatory, antioxidant, and antimicrobial activities have been found in *T. cuspidata*, and they have been used to treat diabetes and high blood pressure [3]. This is because it contains large amounts of active ingredients, such as flavonoids, lignans, steroids, phenolic acids, sesquiterpenes, and glycosides. In addition, *T. cuspidata* has been reported to contain, in particular, 150 taxane compounds [3]. Among these taxanes, paclitaxel was considered to be the most important compound due to its potent and broad-spectrum anticancer effects (such as breast, ovarian, non-small cell lung, Kaposi’s sarcoma, head and neck cancers); moreover, it has gained attention with increasing market demand [1,4]. However, as a secondary metabolite, the content of paclitaxel is very low. The traditional paclitaxel production method is to extract it or its precursor directly from tree bark, which requires the felling of a large number of trees, is unsustainable, and is environmentally unfriendly. To overcome this issue, a chemical synthesis method, semi-synthesis method, endophyte method, and the tissue and cell culture of *Taxus* method were proposed in paclitaxel production [5]. But the first three approaches possessing the drawbacks of higher cost [6], unwanted toxic by-products, mutation-prone cells, and low production limited the paclitaxel yields [7]. Among these methods, plant cell and tissue cultures were the most promising approaches due to adjustable medium compositions and environmental conditions, convenient elicitor stimuli, continuous and industrial production with bioreactors, and an easier extraction of paclitaxel [8,9].

The most effective way through which to increase secondary metabolite production is to activate the plant defense system by using biotic and abiotic elicitors [10,11]. Polyethylene glycol (PEG) is a nontoxic and impermeable high molecular weight polymer, and it can increase the osmotic pressure of the medium and cause the most severe water deficit damage to cells [12]. To respond to these changes, the expression of drought-resistant genes, the activity of the enzymes, and the cell division and expansion rate are regulated, which in turn changes the morphology and structure of the plants [13]. The most common change caused by the osmotic pressure was oxidative stress. The features of this process include the increase in the reactive oxygen species and the level of membrane lipid peroxidation. All these oxygen species disrupted the normal plant metabolism, destroyed the cell membrane, and degraded cells. Therefore, plants have evolved to enhance the antioxidant enzyme activities, such as superoxide dismutase (SOD), catalase (CAT), and peroxidase (PO). In addition, the content of various secondary metabolites—like taxanes, flavonoids, and polyphenols—was increased as antioxidant compounds to deal with the oxidative degradation.

Paclitaxel is a hydrophobic substance with cytotoxic properties [14]. Large accumulations in suspension cells inhibit cell growth and affect paclitaxel production [15]. Therefore, there is an urgent need to induce paclitaxel exocytosis without decreasing cellular activity and paclitaxel synthesis capacity. Cyclodextrin (CD) has a hydrophobic core and hydrophilic surface structures that could encapsulate paclitaxel in the culture medium [16]. Furthermore, cyclodextrins are structurally similar to the alkyl-oligosaccharides released from the cell wall during the initial stages of fungal infections, and they are capable of activating defense responses [17]. As a signaling molecule, salicylic acid (SA) has been shown to regulate physiological processes. Since secondary metabolite accumulation is a common response to stress, SA mediates cell response to stress by inducing defense responses [18], and increases the production of secondary metabolites [19,20]. 

The induction effects of a combination of elicitors significantly improving secondary metabolites have been demonstrated in suspension cell cultures of different species, including *Artemisia annua*, *Taxus media*, and *Vitis vinifera* [21,22]. However, few studies have investigated the physiological responses of *T. cuspidata* suspension cells after treatment with PEG, CD, or SA (alone or in combination). In the present study, the growth, relative electrical conductivity, hydrogen peroxide (H_2_O_2_), malondialdehyde (MDA), superoxide dismutase (SOD), catalase (CAT), peroxidase (PO), phenylalanine ammonia-lyase (PAL), polyphenol oxidase (PPO), soluble sugar, soluble protein, and paclitaxel levels in *T. cuspidata* suspension cells were analyzed under different elicitor treatments. Moreover, the gene expression content of suspension cells (with or without PEG + CD + SA treatment) was measured. According to the KEGG pathway enrichment analysis, the transcriptomics results of the differentially expressed genes (DEGs) involved in paclitaxel biosynthesis were verified with quantitative real-time PCR (RT-qPCR) technology. In addition, the key DEGs related to antioxidant enzymes, paclitaxel synthesis enzymes, and paclitaxel synthesis transcription factors after induction were screened. We clarified the connection between the key DEGs mentioned above and paclitaxel synthesis. Our results were intended to provide a theoretical and experimental basis for industrial paclitaxel production in *T. cuspidata* suspension cells.

## 2. Materials and Methods

### 2.1. Plant Material and Callus Induction

Young and healthy stem nodes with axillary buds (sub-apical) were collected from Changbai mountain (Jilin Province, China) as explants. Stem nodes with axillary buds were trimmed into 10 × 0.2 cm measurements. The explants were washed with detergent and cleaned with running water for 3 h. To remove the surface bacteria, the explants were firstly sterilized with 75% ethanol for 30 s and rinsed with sterile water; they were then subsequently sterilized by a 0.1% mercury chloride solution for 7 min and rinsed with sterile water at least 5 times. The explants were cut into 1 cm sizes and inoculated in a B5 solid medium (Table S1). After inoculation, the culture bottles were placed in an artificial climate incubator (H26442, Beijing Heng Ode Instrument Co., LTD) for 28 days for callus induction. The humidity, temperature, and light conditions were set as 70 ± 5%, 25 ± 1 °C, and dark, respectively. After several subcultures, the homogeneous calli were obtained. For the suspension cell culture, 3 g of the calli were uniformly dispersed into a 100 mL Erlenmeyer flask with 30 mL of a B5 liquid medium (without agar). The temperature and rotational speed of the suspension culture were set as 25 ± 1 °C and 110 rpm. All cultures were grown in darkness.

### 2.2. Establishment of Suspension Cell Culture and Treatment

Homogeneous *T. cuspidata* suspension cells were obtained from several subcultures. To minimize the impact of inoculation, the suspension cell cultures were first pooled together and then equally distributed to obtain each sample. With the single-factor experiments and the response surface optimization tests, the best concentrations of elicitor PEG, CD, and SA for paclitaxel production in *T. cuspidata* suspension cells were 3.6%, 47.5 mmol/L, and 14 mg/L, respectively (Tables S2–S4). The groups of experimental treatments were divided as shown in Table 1. After one day and five days of exposure to the elicitors, respectively, the suspension cells were harvested for analysis. Each 100 mL Erlenmeyer flask was considered as an experimental unit and the experiment was conducted with quintuplicates per treatment. In total, eight flasks were considered as experimental units in each replicate.

### 2.3. Suspension Cell Growth and the Relative Electrical Conductivity

The suspension cells were separated by vacuum filtration with a Brinell funnel and were then weighed (FW). They were then freeze-dried until a constant weight (DW) was achieved. The relative electrical conductivity was conducted according to the method of Chen et al. [23]. Then, 0.1 g of fresh suspension cells was added with 15 mL of sterile distilled water, and these were next placed in a constant temperature water bath at 37 °C for 1 h. Subsequently, the electrical conductivity S1 (DDS-307A, INESA Scientific Instrument Co., Ltd., Shanghai, China) was recorded. Subsequently, the suspension cells with water were put in a hot water bath at 100 °C for 15 min and the electrical conductivity S2 was recorded. The calculation formula is presented below:

$$Y=\frac{S_{1}-S}{S_{2}-S}\times 100$$

where *Y* is the relative electrical conductivity (%), *S*_1_ is the electrical conductivity at 37 °C, *S*_2_ is the electrical conductivity at 100 °C, and *S* is the electrical conductivity of the sterile distilled water.

### 2.4. Hydrogen Peroxide (H_2_O_2_) Determination

The concentration of H_2_O_2_ was measured according to the method of Cao [24]. Next, 0.2 g of suspension cells were ground with 2 mL of 0.1% trichloroacetic acid (TCA) in a pre-cooled mortar. After centrifuging at 4 °C under 1000× *g*, 0.5 mL of supernatant was added with 0.5 mL of phosphate buffer (10 mmol/L, pH 7.0) and 1 mL of KI (1 mol/L), which reacted for 5 min in darkness. The absorbance of the solution was recorded at 390 nm. The H_2_O_2_ concentration was calculated according to the standard curve.

### 2.5. Malondialdehyde (MDA) Determination 

The lipid peroxidation of the suspension cells is normally expressed as the amount of MDA, and this was determined according to the method of Zhang [25] with a thiobarbituric acid (TBA) reaction. Briefly, 0.3 g of the suspension cells were ground with 1 mL of phosphate buffer (10 mmol/L, pH 7.0) in an ice bath. After centrifuging, 0.2 mL of the supernatant was added with 0.6 mL of 5% TCA and 0.2 mL of 0.67% TBA. This was then mixed well, and a reaction took place when under 95 °C for 30 min; next, it was put into an ice bath to terminate the reaction. The amount of MDA (*C*, μM) was calculated by the formulation shown below:

$$C=6.45\times (A_{532}-A_{600})-0.56\times A_{450}$$

### 2.6. Enzyme Activities Determination

For the enzyme extraction, 0.5 g of suspension cells were ground in phosphate buffer (50 mmol/L, pH 7.0) containing 1 mM of EDTA and 1% PVP. The supernatant was carried out to determine the enzyme activities after centrifuging.

#### 2.6.1. Superoxide Dismutase (SOD)

The SOD was carried out according to the nitrogen blue tetrazole method of Salehi et al. [21]. The reaction solution consisted of 3 mL of phosphate buffer (50 mmol/L, pH 7.0), EDTA (0.1 mM), 75 µL of nitroblue tetrazole (NBT), methionine (13 mM), 2 µM of riboflavin, and 100 µL of the enzyme solution. All of the solutions were mixed well and placed 30 cm below the light source, which was composed of four 20 W fluorescent lamps, (Phillips) for 20 min. The solution absorbance at 560 nm was recorded. One unit of SOD activity was described as the enzyme content that inhibited 50% of the NBT photoreduction.

#### 2.6.2. Catalase (CAT)

Hydrogen peroxide has a strong absorption at 240 nm, and this was decomposed by the catalase in this study. Therefore, the catalase activity could be measured according to the absorbance change. Next, 3 mL of phosphate buffer (50 mmol/L, pH 7.0), 2 mL of distilled water, and 0.5 mL of enzyme solution were mixed well, and 0.6 mL of H_2_O_2_ (0.1 mol/L) was added to trigger the reaction. The absorbances at 240 nm were recorded every 10 s until 3 min.

#### 2.6.3. Guaiacol Peroxidase (PO)

Guaiacol can be oxidized to brown products under hydrogen peroxide that is catalyzed by guaiacol peroxidase. These brown products have a strong absorption at 470 nm. According to the method of Sarmadi et al. [21], the reaction mixture consisted of 3 mL of phosphate buffer (50 mmol/L, pH 7.0), 1 mL of H_2_O_2_ (2%), 1 mL of guaiacol (50 mmol/L), and 0.5 mL of enzyme solution. The mixture solution reacted at 37 °C for 15 min, and was then placed into an ice bath with 2 mL of TCA (20%) added immediately to shut down the reaction. The supernatant absorbance was recorded at 470 nm after centrifugation. 

#### 2.6.4. Phenylalanine Ammonia-Lyase (PAL)

The PAL activities were quantified according to the method of Salehi et al. [21]. The reaction mixture included 2 mL of L-phenylalanine (20 mmol/L), 4 mL of boric acid buffer (0.1 mol/L, pH 8.8), and 0.4 mL of enzyme solution. The reaction solution absorbance at 290 nm was recorded at first and then recorded again after incubation at 30 °C for 30 min. 

#### 2.6.5. Polyphenol Oxidase (PPO)

The reaction mixture consisted of 4 mL of phosphate buffer (50 mmol/L, pH 7.0), 2 mL of catechol (0.1 M), and 1 mL of the enzyme solution [21]. The reaction solution absorbances at 410 nm were recorded before and after incubation at 25 °C for 5 min.

### 2.7. Measurement of Soluble Sugar

According to the method of Cao [24], the soluble sugar was qualified by anthrone colorimetry. Briefly, 0.2 g of the suspension cells with 2 mL of buffer were ground in the mortar, and incubated in boiling water for 60 min. After cooling to room temperature, the mixture solution was centrifugated to obtain the supernatant. The mixture of 0.2 mL of the supernatant, 0.8 mL of absolute ethyl alcohol, and 5 mL of anthrone reagent was boiled for 15 min, whereby the absorbances were recorded at 620 nm.

### 2.8. Measurement of Soluble Protein

The Coomassie brilliant blue method was employed to determine the soluble protein. Then, 0.2 g of suspension cells were ground with 2 mL of phosphate buffer (50 mmol/L, pH 7.0) and transferred to a 5 mL volumetric flask. This was then diluted with phosphate buffer to 5 mL. After centrifugation at 4 °C, the supernatant was a crude enzyme solution. The reaction mixture included 0.1 mL of the crude enzyme solution with 5 mL of Coomassie brilliant blue dye, which was then left standing for 5 min to record the absorbances at 595 nm. The amount of soluble protein was calculated by the standard curve.

### 2.9. Paclitaxel Extraction and HPLC Analysis

The freeze-dried suspension cells were first treated with cold plasma (SY-DT02S, Ops Technology Co., Ltd., Suzhou, China) to enhance the surface roughness. The plasma power, gas flow, treatment time, and loading weight were set as 310 W, 120 mL/min, 205 s, and 1 g, respectively. Secondly, the suspension cells (1 g) were mixed with 60 mL of dichloromethane-ethyl alcohol (1:1, *v*/*v*), which were then treated by the high-intensity pulsed electric field under an electric field strength of 16 kV/cm, a pulse number of 8, and a flow rate of 7 mL/min. The supernatant was collected after centrifugation at 8000 r/min. The dichloromethane-ethyl alcohol evaporated and the residues were redissolved in methanol for subsequent HPLC analysis. As per the method of Zhao et al. [26], a Waters e2695 with a C18 reverse-phase column was employed to quantify the paclitaxel. The water (Solvent A) and acetonitrile (Solvent B) were used in a gradient system with a flow rate of 1 mL/min, an injection volume of 10 μL, a column temperature at 30 °C, and a detection wavelength at 227 nm. The gradient elution procedure was set as follows: 40 to 50% B (1–10 min), 50 to 53% B (10–13 min), 53 to 73% B (13–25 min), 73 to 40% B (25–27 min), and 40% B (27–30 min). The standard substance of the paclitaxel brought from Shanghai Yuanye Bio-Technology Co., Ltd. (Shanghai, China) was employed to plot the calibration curves and calculate the paclitaxel concentration in the suspension cells. All injections were repeated three times (*n* = 3). The HPLC chromatograms of paclitaxel standard and paclitaxel production by *T. cuspidata* suspension cells under elicitor treatments are shown in Figure S1.

### 2.10. Transcriptome Sample Preparation and Sequencing

For the transcriptomics analysis, the suspension cells with or without elicitors (PEF, CD, and SA combination) of the T and C group were collected after 1 d incubation. The EasyPure Plant RNA extraction kit (TransGen Biotech Co., Ltd., Beijing, China) was employed to extract the total RNA of the suspension cells. The concentration, purity, and integrity of the RNA were tested by a NonoDrop 2000 spectrophotometer (Thermo Fisher Scientific, Wilmington, DE, USA), agarose gel electrophoresis, and an Agilent Bioanalyzer 2100 system (Agilent Technologies, Santa Clara, CA, USA), respectively. Then, the mRNA was purified from the total RNA using poly-T oligo-attached magnetic beads. The divalent cations were employed to fragment the RNA and the first strand cDNA and second strand cDNA were synthesized using a random hexamer primer with M-MuLV Reverse Transcriptase and DNA Polymerase I with RNase H, respectively. After the adenylation of 3′ ends of DNA fragments, the cDNA fragments obtained from the previous step were amplified by PCR, and the products were purified by an AMPure XP system. The double-stranded PCR products from the previous step were heated, denatured, and circularized by the splint oligo sequence to obtain the final library. The single-stranded DNA was formatted as the final library after cluster generation. RNA-seq was performed by Nanjing Genepioneer Biotechnologies (Nanjing, China).

Differential expression analysis was performed using the DESeq2 package (v.1.26.0). Genes with the threshold |log2(fold change)| > 1, a *p*-value of <0.05, and a Q-value of <0.05 were identified as differentially expressed [27]. GO enrichment was performed with GOseq v2.0. KOBAS software was employed to test the statistical enrichment of the differentially expressed genes (DEGs) in the KEGG pathways. Each treatment contained three biological replicates.

### 2.11. Real-Time qPCR (RT-qPCR) Analysis

The RT-qPCR experiment was measured according to Zhang et al. [28]. The cDNA templates used for RT-qPCR were synthesized with the PrimeScript^TM^ RT reagent Kit for the qPCR synthesis (Takara Biomedical Technology, Dalian, China). RT-qPCR was performed using the FQD-96A Real-Time PCR Detection System (Bioer, Hangzhou, China). The reaction mixture was prepared using the ChamQ SYBR qPCR Master Mix kit (Vazyme Biotech Co., Ltd., Nanjing, China). Ten DEGs that were functionally associated with paclitaxel biosynthesis were selected and validated by RT-qPCR. The primers were designed using Primer Premier 5.0, and the primer sequences are listed in Table S5. The relative expression levels of these genes were normalized by the internal control of TBC41 using the 2^−ΔΔCt^ method.

### 2.12. Statistical Analysis

All the experiments were repeated at least three times. The data were subjected to an analysis of variance (ANOVA) and Duncan’s multiple range test using IBM SPSS Statistics 25.0 (IBM, New York, NY, USA), and these were then expressed as the mean ± standard deviation. Origin 2019 software was employed for mapping. 

## 3. Results and Discussion

### 3.1. Growth Changes

**Figure 1:**
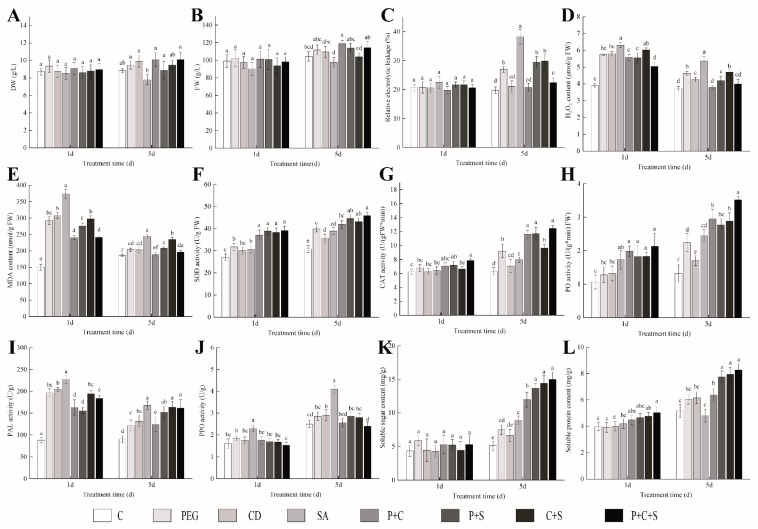
Dry weight (DW) (**A**), fresh weight (FW) (**B**), relative electrolytic leakage (**C**), hydrogen peroxide content (H_2_O_2_ content) (**D**), malondialdehyde content (MDA content) (**E**), superoxide dismutase activity (SOD activity) (**F**), catalase activity (CAT activity) (**G**), guaiacol peroxidase activity (PO activity) (**H**), phenylalanine ammonia-lyase (PAL activity) (**I**), polyphenol oxidase (PPO activity) (**J**), soluble sugar content (**K**), and soluble protein content (**L**) under different elicitor treatments in *T. cuspidata* suspension cells after 1 d and 5 d. The results represent the mean ± standard deviation (SD) error. Different letters indicate statistically significant differences according to Duncan’s multiple range test (*p* < 0.05).

The type and quantity of elicitors could affect the cell growth and paclitaxel production. The DW, FW, and relative electrical conductivity of the suspension cells with different elicitor treatments are shown in Figure 1A–C. For the DW and FW, there were no significant differences between the Control and treatment groups whatever the elicitor type used (*p* > 0.05) (except for the FW of P + C treatment after 5 days of induction). For the PEG-containing treatment groups, the insignificant difference in DW and FW compared to the Control group may be related to the structure of PEG. PEG is a synthetic polymer, which could form stable polymers with toxic phenolic compounds in the cytoplasm, leading to an increase in fresh and dry weights [10]. For the CD-containing treatment groups, CD is able to trap paclitaxel and other secondary metabolites and remove them from the active cytoplasm so that their toxicity does not affect cell viability. This is attributed to the hydrophilic outer surface and hydrophobic mid-cavity structure of CD and its ability to activate ABC transporter proteins [16]. For the SA-containing treatment groups, insignificant difference in DW and FW compared to the Control group may be related to the lower addition level [29]. Notably, after 5 days of induction, there were significant differences in the DW and FW among the seven elicitor treatment groups (except for Control treatment) (*p* < 0.05). The lowest DW and FW values were shown in the SA treatment. Moradi et al. [30] also found a decrease in the weight of the saffron crocus suspension cells with SA added. Significantly, the higher DW and FW of the P + S and P + C + S groups were detected compared with the SA treatment (*p* < 0.05), indicating that the combination elicitor treatments reduced the negative effect of a single elicitor on cell growth. Other scholars have shown that SA with biotic and abiotic stresses enhanced the plant resistance and survival ability [31,32,33], which were in agreement with our results.

The change of suspension cells’ relative electrical conductivity was measured to assess the membrane potential of the plant cell and judge whether the cell membrane has undergone damage after elicitor addition [23]. There was no significant difference in the relative electrical conductivity of the suspension cells after a 1 d treatment (*p* > 0.05), as indicated in Figure 1C. This suggested that *T. cuspidata* suspension cells’ growth would be unaffected by elicitors during a short treatment time. Some scholars have observed that Ginkgo cells could regulate homeostasis between K^+^, Ca^2+^, Mg^2+^ and Na^+^ ions to balance the osmotic pressure [23]. With the time prolonged to 5 d, the PEG, SA, P + S, C + S and P + C + S treatment groups increased the relative electrical conductivity significantly compared with the Control group (*p* < 0.05). These results can be explained by an enhanced membrane permeability of suspension cells [29]. The highest value was observed in the SA group. This may be because the SA stimulated the cells releasing signals (reactive oxygen, etc.) and activated a programmed death [34]. When the relative electrical conductivity among elicitor treatment groups (except Control treatment) was compared, the relative electrical conductivities of the P + S, P + C, and P + S + C groups were significantly lower than the SA treatment group (*p* < 0.05). These phenomena revealed that the combination elicitor treatments decreased the toxic effect of a single-elicitor treatment, and that the number of viable cells increased [35]. 

### 3.2. H_2_O_2_ and MDA Content

The content of H_2_O_2_ and MDA indicated the oxidative stress degree of the plants with elicitor and environment stress [34]. Under these conditions, the oxygen in certain plant enzymes acquired the hydrogen produced by the fatty acid dehydrogenation during the β-oxidation process, and this was then eventually converted to H_2_O_2_ [36]. The main function of H_2_O_2_ at low or normal concentrations are transmitted signals [37], while the high concentration of H_2_O_2_ accelerated lipid peroxidation and caused oxidative damage by accelerating the Haber–Weiss reaction and forming hydroxyl free radicals [38]. Many kinds of research have proved that elicitors cause the oxidative burst of cells (where reactive oxygen species (ROS) are generated quickly and briefly), such as fungi, methyl jasmonate, ultrasound, heat, and shear forces [39]. The content of H_2_O_2_ and MDA after elicitor treatments are shown in Figure 1D,E. All the elicitor treatment groups had a higher H_2_O_2_ content compared with the Control (C) group (*p* < 0.05) after a 1 d treatment, possibly owing to oxidative burst of cells. The highest value was observed in the SA treatment (6.30 μmol/g FW), which was about 1.61 times higher than that of the Control group (C), while the lowest value among elicitor treatment groups (except for the Control group) was obtained at the P + C + S treatment group (*p* < 0.05). After 5 days of induction, the H_2_O_2_ content of the elicitor treatments remained significantly higher than the Control group (*p* < 0.05), except for the P + C and P + C + S groups. Among seven elicitor treatment groups (except for the Control group), the order of H_2_O_2_ content was shown as follows: SA > C + S > PEG > CD > P + S > P + C + S > P + C. The higher H_2_O_2_ content in the individual elicitor treatments was observed compared with the combination inductions. The above results indicated the reduction in the cells’ oxidative damage with the combination elicitor treatments, which was consistent with what was reported by Salehi et al. [21]. As a whole, the H_2_O_2_ content after 5 days of induction was lower than that after 1 day of induction, thus indicating the suspension cells developed an adaptive stress and resistance ability to alleviate the damage. 

MDA is commonly used to measure the degree of cellular membrane lipid peroxidation as a membrane lipid peroxidation production. Under adverse circumstances, the balance between the production and scavenging of free radicals in the plant cells was broken down. The large number of free radicals triggered membrane lipid peroxidation, thereby resulting in MDA accumulation. The MDA contents after different elicitor treatments are shown in Figure 1E. After 1 d of induction, all of the elicitor treatment groups had significantly higher MDA content compared with the Control group (*p* < 0.05). The order of MDA contents is shown: SA > CD > C + S > PEG > P + S > P + C + S > P + C > C. The MDA content of the single-elicitor treatment groups was higher than that of the combination elicitor treatment groups. After 5 days of induction, the MDA content with elicitor treatments remained higher than the Control group, except for the P + C treatment. The highest MDA value was observed after treatment with SA alone, which was a 0.32-fold increase over the Control group, thus suggesting a large number of generated ROS. This phenomenon agrees with the findings of Yu et al. [40]. Similar to the results of 1 day of induction, the lowest MDA content was obtained in the P + C treatment group, followed by the P + C + S treatment, thereby indicating that a combination of elicitors could protect the cell membrane. The reason behind these phenomena may be related to the activation of defense genes and antioxidant enzymes [31,41]. This is meaningful for the industrial production of secondary metabolites with less cell destruction. In *Ctenanthe setosa* cultivation, the results of cell oxidative damage, H_2_O_2_ content, and membrane degradation after SA following a drought treatment were the same as in our research [42]. Similar to the H_2_O_2_ content results, the MDA contents after 5 days of induction were lower than that of 1 day of treatment with an adaptation to the stress environment of cells, except for in the Control group.

### 3.3. Antioxidant Enzyme Activity

Plants have a variety of defense responses, and the antioxidant enzymatic system is one of the main mechanisms for scavenging free radicals to reduce oxidative damage [43]. The activation of antioxidant enzymes (SOD, CAT, and PO) with different elicitor treatments is shown in Figure 1F,G.

For the SOD, all the elicitor treatments, except from the CD group, increased the SOD activity after a 1 d induction (*p* < 0.05), as indicated in Figure 1F. There was no significant difference in the SOD activity in the single-elicitor treatments (PEG, CD, and SA treatment groups), and similar results were obtained among the combination elicitor treatment groups (P + C, P + S, C + S, and P + C + S treatment groups). Notably, the combination elicitor treatments were more effective than single-elicitor treatment in increasing SOD (*p* < 0.05). After 5 days of induction, the highest SOD activity was recorded in the three-elicitor combination group (P + C + S), which was 1.49 times more than the Control. Sarmadi et al. [39] reported similar results in their research. The order of SOD activity was as follows: P + C + S > P + S > C + S > P + C > PEG > SA > CD > C. For the SOD to improve further under a combination elicitor treatment, other scholars have found the same phenomena in an SA treatment with cold stress-treated wheat [44]. SOD is the first and most important enzyme in ROS detoxification. It converted the superoxide radicals (O^2−^) in the cytoplasm, chloroplasts, and mitochondria to H_2_O_2_ to counter the oxidative damage of the free radicals. The H_2_O_2_ produced at this stage was subsequently scavenged by CAT and PO enzymes. The increase in the SOD suggested a cellular defense mechanism against superoxide anions, hydroxyl radicals, and single-linear oxygen species that were generated in response to the elicitors. 

CAT is another antioxidant enzyme that breaks down the H_2_O_2_ into water and oxygen [45]. The CAT content after different elicitor treatments is shown in Figure 1G. There was no significant difference in the CAT content of elicitor treatment groups compared with the Control group after a 1 d induction, except for the P + C + S and P + S treatments. Cao [24] also found insignificant changes in the CAT activity of chrysanthemum suspension cells after 1 day of induction with NaCl. The higher CAT content of P + C + S and P + S treatments proved the activation of defense responses in the suspension cells. After 5 days of induction, a significant increase in the CAT activity following elicitor treatments was observed compared with the Control group (*p* < 0.05), except for the CD treatment. Increased CAT levels indicated the presence of antioxidant defense to destroy, neutralize, or refine free radicals in the plant cells [39]. In other studies, increased CAT and PO activities were also observed with PEG, AgNP-β-CD, and SA treatments [10,31,39,46]. In this study, insignificant changes in the CAT activities of the CD treatment group compared with the Control group after 5 days of induction showed the lower levels of environmental stresses [39]. The degree of oxidative stress may be related to the types and addition levels of CD. 

Peroxidase (PO) has been found in lignin biosynthesis, as well as in biotic and abiotic stress defense. It can scavenge H_2_O_2_ and the mitochondria, as well as reduce oxidative damage. As shown in Figure 1H, the PO activity was increased significantly after a 1 d induction, except for in the CD and PEG treatments. After 5 d of induction, the PO activities of *T. cuspidata* suspension cells all showed a significant increase compared with the Control group (*p* < 0.05). This phenomenon was related to the oxidative stress. The PO activity increasing in our research suggested that H_2_O_2_ and other ROS compounds were actively scavenged by the plant cells, and similar phenomena have been observed in studies by other scholars [24]. In addition, the order of PO activity after 5 d of induction was as follows: P + C + S > P + C > C + S > P + S > SA > PEG > CD > C. This phenomenon suggested that the combination elicitor treatments had higher PO activity compared with the single-elicitor treatment. Similar results have also been reported in many other plants. For example, after a combined induction of callus tissue by SA and glucose, the PO activity was higher than that induced by PEG alone [31]. SA increased PO activity in chickpeas under heat stress [47].

### 3.4. PPO and PAL Activity

Browning is an unwanted phenomenon for plants, and the phenolics were converted by PPO enzyme into harmful substances under elicitors’ treatment [48]. PAL is a key inducible enzyme that links primary and secondary metabolism. Many stress factors (such as temperature, salinity) or elicitors (e.g., SA, methyl jasmonate) can enhance PAL activity [23]. Therefore, it was necessary to regulate the PAL and PPO enzyme activities in the suspension cell cultures.

PAL is one of the earliest committed enzymes in secondary metabolism (the phenylpropionic acid pathway) in plants [49]. It can catalyze the generation of trans-cinnamic acid and ammonium from L-phenylalanine, and it can be considered as a plant resistance biomarker [50]. As shown in Figure 1I, the PAL values were significantly increased after a 1 d induction compared with the Control group (*p* < 0.05), which was related to the defense genes’ expression, the PAL synthesis pathway activation, and the increase in ROS [10,19]. The order of PAL activity after 1 d of induction was as follows: SA > CD > PEG > C + S > P + C + S > P + C > P + S > C. The highest PAL was observed in the SA treatment, which was 2.59 times higher than that of the Control group (C). Similar results were also reported by Rodas-Junco et al. [51,52]. After 5 days of induction, the PAL values of elicitor treatments were significantly higher compared with the Control treatment (*p* < 0.05), thus indicating constant oxidative stress in suspension cells. Notably, the PAL activity of each elicitor treatment group (except for the Control group) after 5 days of induction decreased compared with that of 1 day of induction. Wang [53] also observed a decrease in PAL activity by prolonging the treatment time when treating *Scutellaria baicalensis* suspension cells with PEG, which indicated the adaptation or defense mechanisms exist to combat oxidative stress in suspension cells. These speculations will be further explored in our next study. Since the substrates consumed by PAM and PAL enzymes overlap, the above phenomenon increased the amount of precursor material required for the PAM enzyme reaction and increased the yield of paclitaxel [37].

PPO is the key enzyme for oxidation phenolics in plants, which causes browning and decreases cell viability [54]. In this research, whether induced for 1 day or 5 days, the PPO was increased significantly after the SA treatment compared with the Control group (C) (Figure 1J). After 1 d of induction, there was no significant difference among the other groups (except for the SA treatment). After 5 d of induction, the lowest PPO value was obtained in the P + C + S treatment among all treatment groups, which indicated that the combination elicitor treatments could decrease the PPO activity compared with the single-elicitor induction. As a whole, the PPO value after 5 days of induction was higher than that after 1 day of induction, which may be related to phenolic accumulation. The increased PPO with an SA treatment could be explained by an increasing lignin formation, which formed a barrier to pathogen penetration [45]. Under normal circumstances, phenolic precursors were stored in vesicles; however, under oxidative stress, cellular biofilms were disrupted, and the PPO located in the cytoplasm was able to collide with the precursors, thus leading to browning. However, PEG and CD were added to the medium containing SA, the phenolic compounds generated by the SA induction were enveloped in PEG and CD, which improved the growth and viability of the suspension cells.

### 3.5. Soluble Sugar and Soluble Protein Content

Under stress conditions, plant cells alter their osmotic potential through accumulating osmotic substances like soluble sugars and amino acids to reduce water potential, maintain proper osmotic pressure, and stabilize cell membranes [28,30]. As shown in Figure 1K, there were no significant differences in the soluble sugar content among all of the treatments after a 1 d culture (*p* > 0.05). However, within 5 days of induction, the soluble sugar contents significantly increased with the elicitor treatments compared with the Control group (*p* < 0.05). And the combination elicitor treatment groups (P + C, P + S, C + S, and P + C + S) obtained better effects in improving soluble sugar. The highest value was obtained in the P + C + S treatment, which was 2.93 times higher than that of the Control treatment. The main functions of soluble sugar are the storage of carbon, scavenging free radicals, and the regulation of osmotic pressure. Under stress conditions, polysaccharides are broken down into the low-weight-soluble carbohydrates (sucrose, glucose, fructose, and galactose) [39]. Because the hydroxyl group in soluble sugar can maintain the hydrophilic interactions of proteins like water, soluble sugar with higher concentrations prevented oxidative damage and resisted stress [55]. Other scholars have also reported that soluble sugar content after being treated with sucrose and mannitol is significantly higher than that of the mannitol treatment group [39].

Soluble proteins are mainly important enzymes that maintain cell physiology and metabolic processes [24]. In response to stress, soluble protein in plant cells can be accumulated also to maintain the integrity of cell membranes through their stabilization [28]. After a 1 d induction, except for the C + S and P + C + S treatments, there was no significant difference in the soluble protein content among other treatments (*p* > 0.05), as shown in Figure 1L. After 5 d of induction, the soluble proteins were not significantly different (*p* > 0.05) from the Control group with a single-elicitor treatment (PEG, CD, and SA treatments). The significant higher soluble protein contents compared with the Control group were observed after combination elicitor treatments (P + C, P + S, C + S, and P + C + S treatments). The highest value was obtained after the P + C + S treatment, which was 1.6 times higher than that of the Control group. Shaki et al. [55] also reported the higher soluble protein content of *Safflower Carthamus* following an SA and NaCl combination treatment when compared with NaCl treatment alone. As a whole, the soluble protein of all the groups after 5 days of induction was higher than that of a 1 d culture. These phenomena may be due to the time-consuming nature of synthesizing proteins after stress stimulation and signal transmission [24]. In addition, the secretion and accumulation of protease inhibitors increased with time [22].

### 3.6. Paclitaxel Content

**Figure 2:**
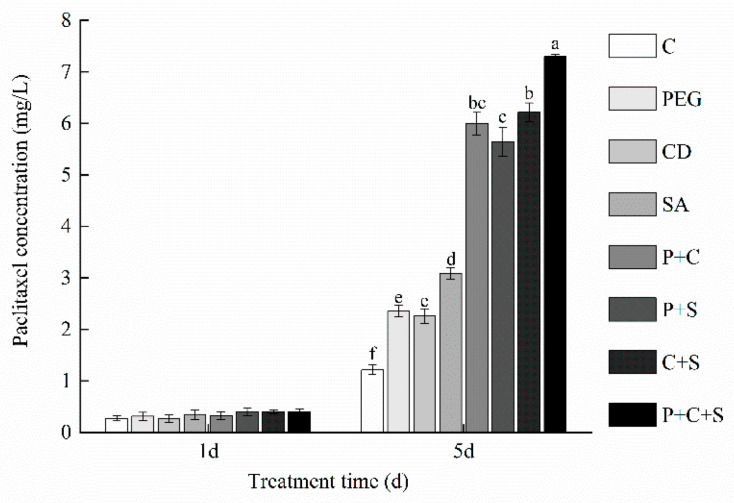
The paclitaxel production of *T. cuspidata* suspension cells under different elicitor treatments. Different letters indicate statistically significant differences according to Duncan’s multiple range test (*p* < 0.05).

Although the exact functions of secondary metabolites are not known, it is generally accepted that they are involved in adverse environmental responses and act as a defense mechanism to establish links between plants and the environment. Paclitaxel is a secondary metabolite of the *T. cuspidata* suspension cell [56]. The paclitaxel contents after the elicitor treatments are shown in Figure 2. There was no significant difference among the treatments after a 1 d culture (*p* > 0.05), which is in agreement with other reports [22]. This was possible because the cells were in the logarithmic growth phase, and the growth dominated at this stage. With the culture prolonged, all the elicitor treatment groups increased the paclitaxel content significantly compared with the Control group. And the paclitaxel yield was 6.02, 5.13, 4.95, 4.65, 2.54, 1.94, and 1.86 times higher than that of the Control group in the P + C + S, S + C, P + C, P + S, SA, PEG, and CD treatment groups, respectively, which suggested that the combination elicitor treatments were more effective than a single-elicitor treatment. These phenomena were such because three elicitors generate different defense response signaling, but they are cross-linked in the signaling pathway and showed synergistic effects in increasing paclitaxel production. Other scholars have treated sequoia healing tissues, suspension cells, or hazelnut cells with CD + methyl jasmonate [57], SA + glucose [30], lauryl alcohol + methyl jasmonate [15], and ultrasound + SA [58], respectively; on this, they found that the paclitaxel yields were significantly higher than those obtained following single treatments. In particular, the paclitaxel yield of the *Taxus Chinese* suspension cells with SA and fungal elicitors was 1.5 times, 2.3 times, and 7.5 times higher than that of the fungal elicitor treatment group, SA treatment group, and the Control group, respectively [40]. In addition to paclitaxel, the accumulation of other secondary metabolites (e.g., medicinal alkaloids) was also significantly promoted by the combination elicitor treatments [56]. These reports above were consistent with the results of the present study.

### 3.7. Transcriptional and Functional Enrichment Analysis

**Figure 3:**
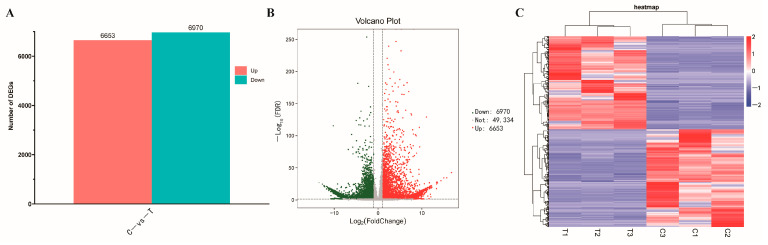
The differentially expressed genes (DEGs) in *T. cuspidata* suspension cells respond to the elicitor treatments. (**A**) Statistics of the number of DEGs between groups. Red and blue histograms represent the number of significant up-regulation and down-regulation genes. (**B**) The volcanic map of DEGs. (**C**) Clustering heat map of the DEG expressions.

To reveal the paclitaxel biosynthesis mechanism under induction stress, the transcriptomes of *T. cuspidata* suspension cells with and without elicitor treatments were compared. The Control group was set as C, and the P + C + S treatment group was set as T. After RNA-seq, each sample had 6.94 Gb clean reads. The value of Q20 and Q30 for all samples exceeded 90%, thus meeting the sequencing requirements (Table S6). A total of 13,623 differentially expressed genes (DEGs) were identified after different treatments (with and without elicitors) (Figure 3).

**Figure 4:**
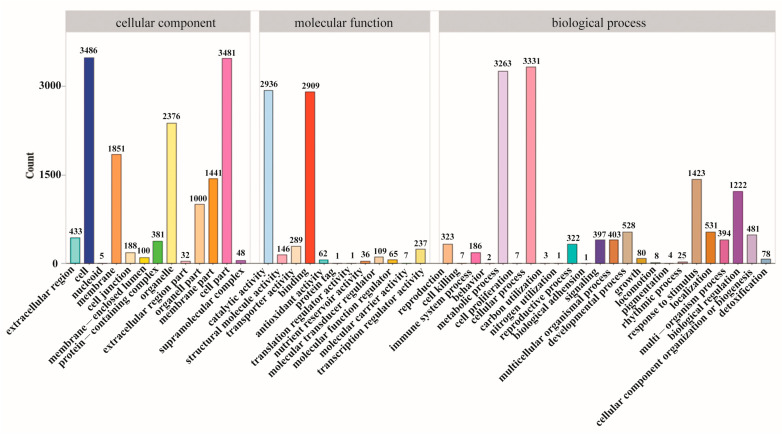
GO functional analysis of the differentially expressed genes (DEGs) with elicitor treatments.

**Figure 5:**
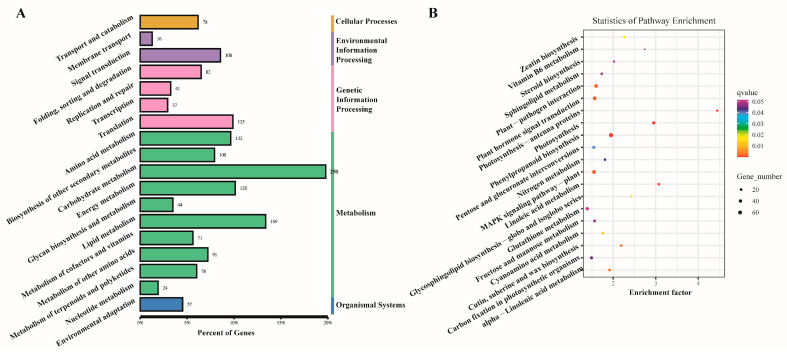
KEGG pathway analysis of the differentially expressed genes (DEGs) with elicitor treatments. (**A**) KEGG pathway classification chart of the DEGs. (**B**) KEGG pathway enrichment analysis bubble chart of the DEGs.

Gene ontology (GO) enrichment analysis was performed to describe the differences in gene function. As shown in Figure 4, the DEGs affected a total of 13 cellular component units, 12 molecular function units, and 24 biological process units. In the biological process units, the metabolic process was strongly influenced by the elicitor treatments. No function of an organism can be realized without the coordinated action of different gene products. Pathway annotation analysis of differentially expressed genes by the Kyoto Encyclopedia of Genes and Genomes (KEGG) database could help with a further deciphering of gene functions. As shown in Figure 5, 1619 DEGs were annotated in the 18 pathways. The DEGs were mainly enriched in terms of plant–pathogen interactions, plant hormone signal transduction, photosynthesis, phenylpropanoid biosynthesis, and in MAPK signaling pathway/plant pathways.

### 3.8. Differential Expression of Antioxidant Enzyme Genes

In the previous experiments, the contents and activities of H_2_O_2_, MDA, and antioxidant enzymes (SOD, CAT, PO) were significantly increased after elicitor induction, and we hypothesized the up-regulation expressions of antioxidant enzyme genes with oxidative stress. To verify the inferences, the DEGs related to the antioxidant enzyme were analyzed, and eight up-regulated genes controlling POD, two up-regulated genes controlling SOD, and one up-regulated gene controlling CAT were identified (Table 2). Therefore, it could be inferred that the expression of SOD, CAT, and PO enzyme genes, as well as the content of antioxidant enzymes, were increased in *T. cuspidata* suspension cells under elicitor-stimulated oxidative stress, which in turn promoted the synthesis and secretion of the secondary metabolite paclitaxel.

### 3.9. Differential Expression of Enzyme Genes in Paclitaxel Synthesis Pathway

**Figure 6:**
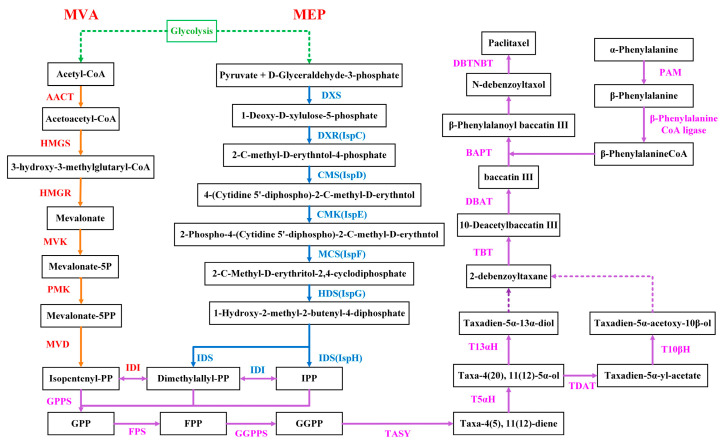
The synthesis pathway of paclitaxel.

The paclitaxel biosynthesis pathway in the *T. cuspidata* suspension cells was mapped based on relevant reports and KEGG annotation results (Figure 6). The MEP and MVA pathways were the basis for paclitaxel synthesis. The DEGs of the key enzymes in paclitaxel synthesis are shown in Table 3. Furthermore, it was hypothesized that the elicitors promoted the paclitaxel biosynthesis by regulating the gene expression of the key enzymes in the paclitaxel synthesis pathway.

### 3.10. Differential Expression of Transcription Factors in Paclitaxel Synthesis Pathway

**Figure 7:**
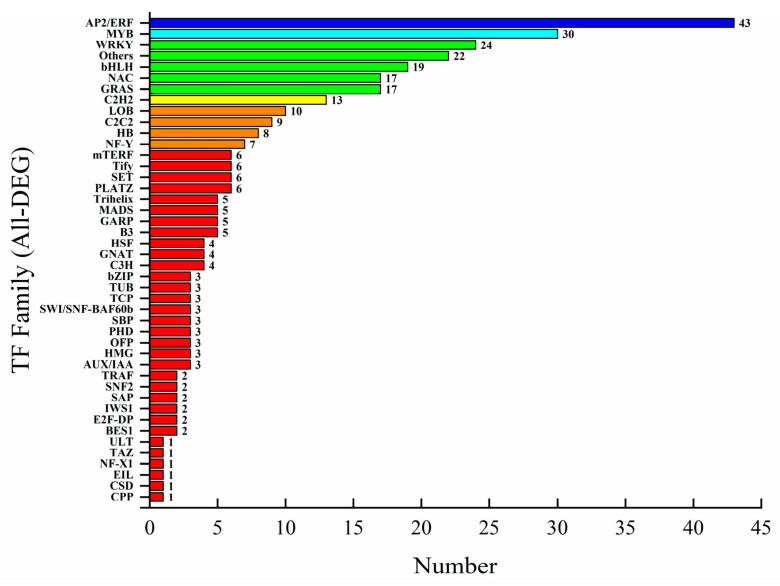
The chart of differentially expressed transcription factors.

Transcription factors are important components of plant responses to environmental changes and in the stimulation of cellular defense responses. As a secondary metabolite, the synthesis of paclitaxel was regulated by transcription factors, such as ERF, MYB, WRKY, and bHLH. Some scholars have proved the correlation between the expression of transcription factors (ERF, MYB, WRKY, and bHLH) in paclitaxel biosynthesis [59]. And the positive regulations of TFs such as MYC2, JAMYC, WRKY, ERF, TIFY, R2RE, MYB, and bHLH on increasing expression of paclitaxel synthesis genes in *Taxus* cell cultures under MeJA induction were also reported in the research of De Geyter et al. [60]. In this study, there were 319 transcription factors that were differently expressed after the elicitor treatments, as shown in Figure 7. The transcription factor family with the highest number of DEGs was the ERF family, followed by the MYB, WRKY, bHLH, GRAS, and NAC families. Other scholars have proved that tropane and paclitaxel synthesis were regulated by the ERF and WRKY families [61,62].

### 3.11. Real-Time qPCR Validation

**Figure 8:**
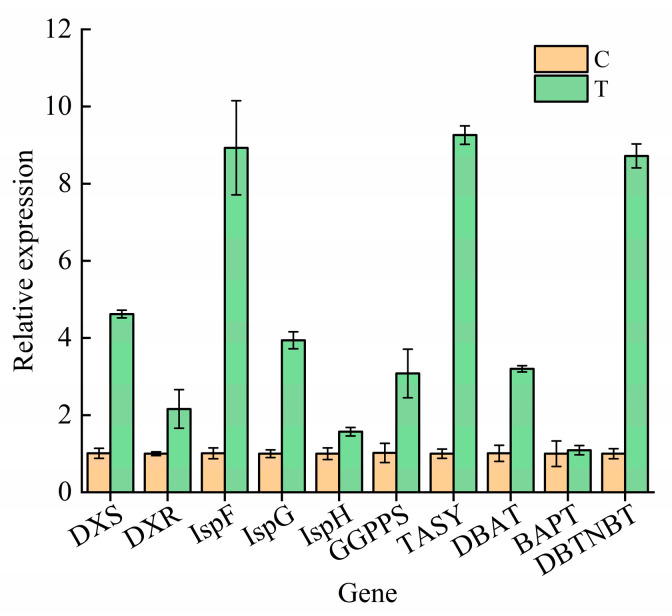
Relative expression of the 10 differentially expressed genes (DEGs) involved in paclitaxel synthesis between normal conditions (C) and elicitor P + C + S treatments (T).

To verify the DEGs of the *T. cuspidata* suspension cells after the elicitor treatments, 10 key enzyme genes of paclitaxel synthesis were selected to determine the expression level by the real-time qPCR technology according to the paclitaxel synthesis pathway, as shown in Figure 8. The expression levels of all candidate genes were up-regulated, which was consistent with the transcriptome results, thereby indicating that the transcriptome sequencing results were reliable. Therefore, we could hypothesize that the increase in paclitaxel yield after the elicitor P + C + S treatments (T) was due to the increased gene expressions of the transcription factors and key enzymes of paclitaxel synthesis.

## 4. Conclusions

The physiological and biochemical reactions of *T. cuspidata* suspension cells after treatment with PEG, CD, or SA (alone or in combination) for 1 day and 5 days were reported for the first time. Induction with elicitors caused oxidative damage in the suspension cells by increasing the relative electrical conductivity and raising the content of H_2_O_2_ and MDA. In response to the oxidative damage, the defense response was activated, along with an increase in the antioxidant enzyme activities and paclitaxel production. The combination elicitor treatments also enhanced the antioxidant enzyme activities and the levels of soluble sugar and soluble protein. A decreasing content of H_2_O_2_ and MDA and lower activities in PAL and PPO were similarly observed compared with the single-elicitor treatment. The tolerance of suspension cells was improved, while the paclitaxel yield was increased after induction with the combination elicitor treatments. The DEGs associated with paclitaxel synthesis were identified. The regulatory mechanism of combination elicitor treatments on improving paclitaxel accumulation in *T. cuspidata* suspension cells was preliminarily revealed. In conclusion, induction with combination elicitor treatments is more effective than single-elicitor treatment in reducing oxidative damage and increasing cell biomass and survival, as well as paclitaxel yields. Furthermore, it provides a convenient and cost-effective method in using suspension cells in industrialized bioreactors for the target compounds’ production.

## Figures and Tables

**Table 1:** The groups of the experimental treatments.

Group	PEG (%)	CD (mmol/L)	SA (mg/L)
C	0	0	0
PEG	3.6	0	0
CD	0	47.5	0
SA	0	0	14
P + C	3.6	47.5	0
P + S	3.6	0	14
C + S	0	47.5	14
P + C + S(T)	3.6	47.5	14

**Table 2:** The statistical analysis of the differentially expressed genes (DEGs) associated with superoxide dismutase (SOD), catalase (CAT), and peroxidase (PO).

UniGene ID	Nr Description	Fold Change	Trends
TRINITY_DN10687_c0_g1	Class III secretory peroxidase (*Ginkgo biloba*)	2.1824	Up
TRINITY_DN2234_c0_g1	Putative ascorbate peroxidase (*Cryptomeria japonica*)	1.4879	Up
TRINITY_DN20043_c1_g1	Peroxidase 10-like (*Nicotiana attenuate*)	6.2079	Up
TRINITY_DN16051_c2_g1	Class III secretory peroxidase (*Ginkgo biloba*)	6.3471	Up
TRINITY_DN776_c0_g1	Peroxidase (*Picea abies*)	2.7717	Up
TRINITY_DN6282_c0_g1	Peroxidase 12 precursor, putative (*Ricinus communis*)	2.9376	Up
TRINITY_DN7673_c0_g1	Class III plant secreteperoxidase (*Chamaecyparis obtuse*)	9.2501	Up
TRINITY_DN7673_c0_g2	Peroxidase (*Picea abies*)	2.3967	Up
TRINITY_DN10591_c0_g1	Superoxide dismutase activity	1.1457	Up
TRINITY_DN631_c0_g2	Removal of superoxide radicals	1.1099	Up
TRINITY_DN275_c0_g1	Catalase activity	2.1807	Up

**Table 3:** The differentially expressed genes (DEGs) of the paclitaxel synthesis pathway.

EC	UniGene ID	Fold Change	Trends
HMGR	TRINITY_DN2873_c1_g1	−2.6733	down
MVD	TRINITY_DN3862_c0_g2	1.304	up
DXS	TRINITY_DN2395_c0_g1	3.000	up
	TRINITY_DN11223_c0_g1	−1.1069	down
DXR	TRINITY_DN2533_c0_g1	1.7704	up
IspE	TRINITY_DN22846_c1_g1	3.1831	up
IspF	TRINITY_DN497_c0_g1	2.7632	up
IspG	TRINITY_DN2343_c0_g1	2.1063	up
IspH	TRINITY_DN1245_c0_g1	1.8094	up
GGPS	TRINITY_DN7266_c0_g1	1.1176	up
GGPPS	TRINITY_DN33697_c0_g4	1.8992	up
	TRINITY_DN5982_c0_g1	1.4502	up
TASY	TRINITY_DN9815_c0_g1	10.7548	up
	TRINITY_DN40292_c0_g1	9.3180	up
DBAT	TRINITY_DN887_c0_g2	1.2216	up
	TRINITY_DN1144_c1_g3	3.1702	up
	TRINITY_DN6595_c0_g1	2.6967	up
BAPT	TRINITY_DN1024_c0_g1	1.1482	up
DBTNBT	TRINITY_DN109516_c0_g1	9.0914	up
	TRINITY_DN102188_c0_g1	7.8285	up
	TRINITY_DN23180_c0_g1	−1.0779	down
	TRINITY_DN5209_c0_g1	7.0817	up

## Data Availability

Data are contained within the article.

## Supplementary Materials

The following supporting information can be downloaded at the following: https://www.mdpi.com/article/10.3390/plants12223817/s1. Table S1: B5 solid medium composition; Table S2: Dry weight (DW), fresh weight (FW), relative electrolytic leakage and paclitaxel production under PEG, CD, or SA treatment in *T. cuspidata* suspension cells; Table S3: Response surface test design and results; Table S4: The analysis of variance (ANOVA) for response surface polynomial model; Figure S1: HPLC chromatograms of paclitaxel standard (A) and paclitaxel production by *T. cuspidata* suspension cells under elicitor treatments (B); Table S5: Primers for quantitative real-time PCR analysis of genes involved in paclitaxel synthesis and associated with elicitors treatment; Table S6: Statistics of RNA-Seq reads.
