# Supplementary material for: Transcriptomics and Physiological Analyses Reveal Changes in Paclitaxel Production and Physiological Properties in Taxus cuspidata Suspension Cells in Response to Elicitors
Source: Plants (Basel). 2023 Nov 10;12(22):3817. doi: 10.3390/plants12223817 (PMC10674800; doi:10.3390/plants12223817)
Supplement: Supplementary file 1 [file plants-12-03817-s001.zip › plants-2642264-supplementary.pdf]

Supplementary File

Supplementary Table S1

Table S1. B5 solid medium composition.

Nutrient component	Concentration (mg/L)
Sucrose	20000
Agar	7000
KNO_3	3000
Hydrolyzed milk protein	500
$MgSO_4·7H_2O$	500
Inositol	200
$NaH_2PO_4·H_2O$	150
$CaCl_2·2H_2O$	150
$(NH_4)_2SO_4$	134
Na_2EDTA	37.3
$FeSO_4·7H_2O$	27.8
Vitamin B_1	20
$MnSO_4·H_2O$	10
H_3PO_3	3
Vitamin B_6	3
$ZnSO_4·7H_2O$	2
Niacin	2
Glycine	2
α-Naphthalene Acetic Acid	2
KI	0.75
$Na_2MoO_4·2H_2O$	0.25
$CuSO_4·5H_2O$	0.025
$CoCl_2·6H_2O$	0.025

Supplementary Table S2

Table S2. Dry weight (DW), fresh weight (FW), relative electrolytic leakage and paclitaxel production under PEG, CD, or SA treatment in *Taxus cuspidata* suspension cells.

Treatment	DW (g/L)	FW (g/L)	Relative electrolytic leakage (%)	Paclitaxel (mg/L)
1.5% PEG	13.20±0.31	142.19±0.42	29.65±1.43	1.62±0.11
3% PEG	13.81±0.30	150.00±0.59	31.63±1.95	2.55±0.15
4.5% PEG	13.49±0.58	140.38±0.60	49.01±1.76	1.85±0.14
6% PEG	8.09±0.88	119.95±0.63	62.52±1.47	1.63±0.12
25 mmol/L CD	11.99±1.24	147.41±7.56	25.66±1.13	1.73±0.14
50 mmol/L CD	14.98±1.21	171.55±8.25	24.29±1.65	2.40±0.15
75 mmol/L CD	14.07±0.60	157.03±8.18	27.28±2.57	2.30±0.12
100 mmol/L CD	9.62±0.89	142.40±6.57	33.69±2.21	1.95±0.12
5 mg/L SA	11.69±0.31	129.38±7.19	32.52±4.58	2.10±0.10
10 mg/L SA	11.41±0.28	119.01±8.20	43.58±2.74	2.69±0.10
15 mg/L SA	11.11±0.31	115.22±8.38	61.04±5.98	3.20±0.12
20 mg/L SA	10.80±0.29	100.78±5.60	72.34±3.49	3.56±0.15

Supplementary Table S3

Table S3. Response surface test design and results.

	A-PEG (%)	B-CD (mmol/L)	C-SA (mg/L)	Paclitaxel (mg/L)
1	-1 (2)	1 (65)	0 (20)	2.87
2	0 (3)	0 (50)	0	6.92
3	0	-1 (35)	1 (30)	4.18
4	0	1	-1 (10)	5.14
5	0	0	0	7.52
6	1 (4)	0	-1	7.81
7	-1	-1	0	4.83
8	0	0	0	8.11
9	1	0	1	3.99
10	0	0	0	7.67
11	-1	0	-1	4.04
12	0	-1	-1	6.21
13	1	1	0	5.02
14	0	0	0	6.94
15	1	-1	0	6.03
16	0	1	1	3.35
17	-1	0	1	4.00

Supplementary Table S4

Table S4. The analysis of variance (ANOVA) for response surface polynomial model.

Source	Sum of squares	df	Mean square	F value	p-value
Model	45.30	9	5.03	29.54	＜0.0001***
A-PEG	6.32	1	6.32	37.09	0.0005***
B-CD	2.96	1	2.96	17.40	0.0042***
C-SA	7.37	1	7.37	43.27	0.0003***
AB	0.23	1	0.23	1.32	0.2876
AC	3.57	1	3.57	20.97	0.0025***
BC	0.014	1	0.014	0.085	0.7797
A^2	6.60	1	6.60	38.75	0.0004***
B^2	9.38	1	9.38	55.03	0.0001***
C^2	6.26	1	6.26	36.77	0.0005***
Residual	1.19	7	0.17		
Lack of fit	0.16	3	0.055	0.21	0.8827
Pure error	1.03	4	0.26		
Cor total	46.49	16			
R^2=0.9743 AdjR^2=0.9414 C.V.=7.42% AQ=15.166					

*Significant at P < 0.05, ** Significant at P < 0.01, *** Significant at P < 0.001

Supplementary Figure S1

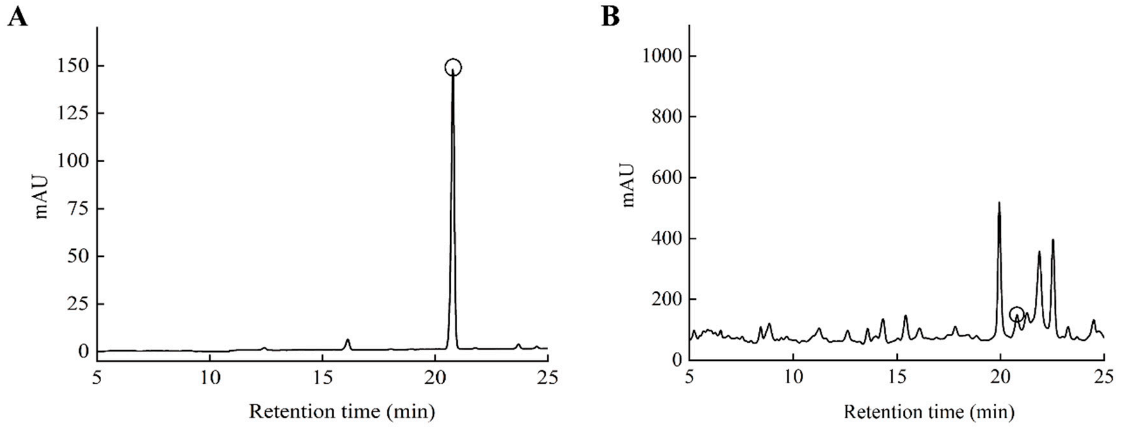

Figure S1. HPLC chromatograms of paclitaxel standard (A) and paclitaxel production by *T. cuspidata* suspension cells under elicitor treatments (B).

Supplementary Table S5

Table S5. Primers for quantitative real-time PCR analysis of genes involved in paclitaxel synthesis and associated with elicitors treatment.

Gene name	Sequence	Product sizes (bp)
TBC41	F: CAAGAAGAAAGAGTCAGCAAATGG<br>R: GGAACGACATGACATTATGAATAGC	91
DXS:<br>TRINITY_DN2395_c0_g1	F: GGGTTTTCGTGTTTATCGGC<br>R: CGTAAGCTTGTCCAGCAGTCAT	156
DXR:<br>TRINITY_DN2533_c0_g1	F: ACACTAACTTGGCACTTTC<br>R: AAGGAGCTCTGCGACGTA	162
IspF:<br>TRINITY_DN497_c0_g1	F: ATGGCGGGATGCTAAAACG<br>R: CTCAATGTGGAGCAAACAAAGA	207
IspG:<br>TRINITY_DN2343_c0_g1	F: TCTCAACTGGTGCCCACGA<br>R: GGGGAAGTTCAGGGCGTTT	212
IspH:<br>TRINITY_DN1245_c0_g1	F: GGCATAGTAGATGGGGGT<br>R: TGATGTGTGCGTGTGAGA	193
GGPPS:<br>TRINITY_DN33697_c0_g4	F: TATCCTTTCCCTTCGCCTTC<br>R: AGAGAATGGCTTACACGGCA	147
TASY:<br>TRINITY_DN9815_c0_g1	F: AGCCGATGATGTCCCGAT<br>R: AGTCCTTAATCTCCTCGTT	143
DBAT:<br>TRINITY_DN1144_c1_g3	F: GATTCCATCGGGGTTATTCTTG<br>R: GGCGATTGGGATTTGATGAAG	143
BAPT:<br>TRINITY_DN1024_c0_g1	F: ATACTCCTCGCCTTGTTTCACC<br>R: GGCTCAGAAGGAAAGAAAATGG	274
DBTNBT:<br>TRINITY_DN109516_c0_g1	F: GAAATGTTGTCAGGGGCA<br>R: AGCGGAATCAGGATGGAG	185

Supplementary Table S6

Table S6. Statistics of RNA-Seq reads.

Sample name	Read Sum	Base Sum	GC (%)	N (%)	Q20 (%)	Cycle Q20 (%)	Q30 (%)
C1	23523922	7058076600	45.21	0.00	97.95	100.00	93.89
C2	25446602	7633980600	44.65	0.00	98.07	100.00	94.14
C3	24828539	7448561700	45.25	0.00	97.86	100.00	93.73
T1	23133225	6939967500	44.74	0.00	98.13	100.00	94.28
T2	27767789	8330336700	44.37	0.00	97.73	100.00	93.36
T3	24226452	7267935600	44.59	0.00	97.87	100.00	93.72
